# Imaging biomarkers for evaluating tumor response: RECIST and beyond

**DOI:** 10.1186/s40364-021-00306-8

**Published:** 2021-07-02

**Authors:** Ching-Chung Ko, Lee-Ren Yeh, Yu-Ting Kuo, Jeon-Hor Chen

**Affiliations:** 1 Department of Medical Imaging, Chi Mei Medical Center, Tainan, Taiwan; 2 Department of Health and Nutrition, Chia Nan University of Pharmacy and Science, Tainan, Taiwan; 3 Department of Radiology, E-DA Hospital, I-Shou University, Kaohsiung, Taiwan; 4 Department of Medical Imaging, Kaohsiung Medical University Hospital, Kaohsiung, Taiwan; 5 Tu & Yuan Center for Functional Onco-Imaging, Department of Radiological Sciences, University of California, 164 Irvine Hall, Irvine, CA 92697 − 5020 USA

**Keywords:** RECIST, Imaging biomarker, Tumor response

## Abstract

Response Evaluation Criteria in Solid Tumors (RECIST) is the gold standard for assessment of treatment response in solid tumors. Morphologic change of tumor size evaluated by RECIST is often correlated with survival length and has been considered as a surrogate endpoint of therapeutic efficacy. However, the detection of morphologic change alone may not be sufficient for assessing response to new anti-cancer medication in all solid tumors. During the past fifteen years, several molecular-targeted therapies and immunotherapies have emerged in cancer treatment which work by disrupting signaling pathways and inhibited cell growth. Tumor necrosis or lack of tumor progression is associated with a good therapeutic response even in the absence of tumor shrinkage. Therefore, the use of unmodified RECIST criteria to estimate morphological changes of tumor alone may not be sufficient to estimate tumor response for these new anti-cancer drugs. Several studies have reported the low reliability of RECIST in evaluating treatment response in different tumors such as hepatocellular carcinoma, lung cancer, prostate cancer, brain glioma, bone metastasis, and lymphoma. There is an increased need for new medical imaging biomarkers, considering the changes in tumor viability, metabolic activity, and attenuation, which are related to early tumor response. Promising imaging techniques, beyond RECIST, include dynamic contrast-enhanced computed tomography (CT) or magnetic resonance imaging (MRI), diffusion-weight imaging (DWI), magnetic resonance spectroscopy (MRS), and ^18^ F-fluorodeoxyglucose (FDG) positron emission tomography (PET). This review outlines the current RECIST with their limitations and the new emerging concepts of imaging biomarkers in oncology.

**Supplementary Information:**

The online version contains supplementary material available at 10.1186/s40364-021-00306-8.

## Introduction

Tumor response assessment is important in clinical trials and standard cancer treatments. Both tumor regression and disease progression detected by imaging biomarkers are important endpoints. Imaging biomarkers can detect the subtle changes in physiology and pathology before their clinical detection and thus act as surrogate endpoints reducing time and resources used in cancer clinical trials [1]. Besides, imaging biomarkers are also used as predictive classifiers to assist in selecting appropriate candidates for particular treatments [2]. Conventionally, morphologic change in tumor size is usually related to survival length and has been considered as a surrogate endpoint of therapeutic efficacy by the World Health Organization (WHO) criteria and Response Evaluation Criteria in Solid Tumors (RECIST 1.0 and 1.1 versions) (Table 1). Currently, RECIST 1.1 is the gold standard for the assessment of treatment response in solid tumors [3, 4]. Traditional chemotherapies are cytotoxic and act primarily by eliminating neoplastic cells. Therefore, a change in tumor size indicates a change in the number of neoplastic cells and has thus evolved into a radiologic biomarker of treatment response [5]. However, unlike cytotoxic drugs, molecular-targeted therapy, and immunotherapy, which have emerged in the past 15 years, can effectively interfere with signaling pathways and thereby inhibit cell growth. For these new treatments, tumor necrosis or lack of tumor progression may be associated with an improvement in outcome, even in the absence of major shrinkage of tumors [6]. Thus, the assessment of morphologic changes by WHO and unmodified RECIST criteria alone may not be sufficient to estimate tumor response in patients who received new targeted therapies [6–8].

**Table 1:** Comparison between WHO, RECIST, and RECIST 1.1 Criteria

	WHO	RECIST	RECIST 1.1
Lesion measurement			
Imaging modalities	N/A	Chest X-ray, CT, and MRI	Chest X-ray, CT, MRI, and ^18^ F-FDG PET
Limitation of measurable lesions	N/A	• 10 mm on spiral CT • 20 mm on non-spiral CT or MRI • Chest X-ray: if clearly defined • Clinical: 20 mm	• 10 mm on CT/MRI (slice thickness ≤ 5 mm), or 2 x slice thickness (if thickness > 5 mm ) • Chest X-ray: 20 mm • Clinical: 10 mm (must be measurable with calipers) ^•18^ F-FDG PET: included only in the detection of new lesions
Lymph node	Unspecified	Unspecified	CT: short axis • ≥ 15 mm is measurable, target lesion • 10–14 mm is non-measurable, non-target lesion • < 10 mm is normal
Method of measurement	Cross-product of the longest diameter and the longest perpendicular diameter	Longest diameter in the axial plane	Longest diameter in the axial plane
Numbers of lesions measured	N/A	up to 10 lesions (≤ 5 in any one organ)	up to 5 lesions (≤ 2 in any one organ)
Response evaluation			
Complete Response (CR)	Disappearance of all lesions	Disappearance of all lesions	Disappearance of all lesions and pathologic lymph nodes
Partial Response (PR)	≥ 50 % decrease in the sum of the area	≥ 30 % decrease in the sum of the longest diameter	≥ 30 % decrease in the sum of the longest diameter
Stable Disease (SD)	Neither PR nor PD	Neither PR nor PD	Neither PR nor PD
Progressive Disease (PD)	≥ 25 % increase in the sum of the area	≥ 20 % increase in the sum of longest diameters, or new lesions	≥ 20 % increase in the sum of longest diameters with an absolute increase of ≥ 5 mm, or new lesions

*CT* computed tomography, *MRI* magnetic resonance imaging, *N/A* not applicable, *PET* positron emission tomography, *RECIST* Response Evaluation Criteria in Solid Tumor, *WHO* World Health Organization

There is an increased need for reliable imaging methods associated with early tumor response considering changes in other tumor characteristics such as tumor viability, metabolic activity, and attenuation. Several studies have reported low reliability of RECIST in evaluating treatment response in different tumors, such as hepatocellular carcinoma (HCC) [9], prostate cancer [10], brain glioblastoma [11], bone metastasis [12], malignant pleural mesothelioma (MPM) [13], and disseminated or ill-defined lymphoma [14]. Therefore, various specific criteria other than RECIST for response assessment of different tumors have been proposed (Table 2) [15]. According to RECIST, the selection of targeted lesions may be different among the readers for patients with multiple lesions. Variability may include scan-rescan and both intra- and inter-observer inconsistency between two repeat readings of the same scan. Tumor size changes between interval studies may arise from true tumor changes and concomitant errors in measurement.

**Table 2:** Summary of major tumor response assessment criteria other than RECIST

Author / Year	Criteria	Brief description
Hepatocellular carcinoma (HCC)		
Lencioni, R. et al. (2010) [16]	modified RECIST (mRECIST)	• To resolve limitations of anatomic tumor response metrics when applied RECIST 1.1 to molecular-targeted therapies or locoregional therapies in HCC. • Reassessment of progression that could be misinterpreted in RECIST 1.1 due to the natural progression of chronic liver disease (ascites, enlargement of lymph nodes, etc.). • Only well-delineated, arterially enhancing lesions can be selected as target lesions. • Number of target lesions: up to 5 lesions (≤ 2 in any one organ). • Short axis of porta hepatis lymph nodes ≥ 20 mm or other lymph nodes ≥ 15 mm are considered as malignant.
Brain tumor		
Macdonald, D.R. et al. (1990) [17]	McDonald	• Using contrast-enhanced CT and MRI scans of the head. • Response assessment is based on changes in tumor size (the product of the maximal cross-sectional enhancing diameters). • Considering the use of corticosteroids and changes in the neurologic status of the patient.
Wen, P.Y. et al. (2010) [18]	RANO	• Response Assessment in Neuro-Oncology (RANO) criteria. • An update to the McDonald Criteria which also takes into consideration of non-enhancing tumor components, and lesions on the T1/T2-weighted and fluid-attenuated inversion recovery (FLAIR) MRI sequences. • Definition of measurability. • Number of target lesions: up to 5 lesions. • Pseudo-progression considered.
Bone metastasis		
Hamaoka, T. et al. (2004) [19]	MDA	• MD Anderson (MDA) Bone Response Criteria. • An approach for diagnosis and assessment of bone metastasis. • Quantitative and qualitative assessments of the behavior of bone metastases based on x-ray, CT, and MRI.
Lymphoma		
Cheson, B.D., et al. (2007) [20]	Revised Cheson	• Definition of standardized response criteria for Hodgkin’s and non-Hodgkin’s lymphoma using ^18^ F-FDG PET, immunohistochemistry, and flow cytometry.
Cheson, B.D., et al. (2014) [21]	Lugano	• Represent a set of revised recommendations regarding the use of the Cheson criteria and Deauville five-point scale, and formally incorporated ^18^ F-FDG PET into standard staging and response evaluation for FDG-avid lymphomas.
Gastrointestinal stromal tumor (GIST)		
Choi, H. et al. (2007) [22]	Choi	• CT criteria for evaluation of response to imatinib therapy in gastrointestinal stromal tumor (GIST). • Combination of tumor size and tumor attenuation on CT (a 10 % decrease in tumor size or a more than 15 % decrease in tumor attenuation at 2 months of treatment) were used. • Defining progressive disease by (1) appearance of new lesions, (2) appearance or increase in size of intratumoral nodules, or (3) tumor size increase by more than 20 % without post-treatment hypodense change.
^18^ F-FDG PET		
Young, H. et al. (1999) [23]	EORTC PET response	• European Organization for Research and Treatment of Cancer (EORTC) PET response. • Proposed a common method of assessing tumor ^18^ F-FDG uptake and reporting of response data.
Wahl, R.L. et al. (2009) [24]	PERCIST	• PET Response Criteria in Solid Tumors (PERCIST). • Qualitative and quantitative approaches to metabolic tumor response assessment with ^18^ F-FDG PET.
Goldfarb, L. et al. (2019) [25]	iPERCIST	• Immune PET Response Criteria in Solid Tumors (iPERCIST). • Monitoring anti-programmed cell death 1 (PD-1)-based immunotherapy in non-small cell lung cancer with ^18^ F-FDG PET.
Immunotherapy		
Wolchok, J.D. et al. (2009) [26]	irRC	• The immune-related Response Criteria (irRC) • Bidimensional (the product of the maximal cross-sectional diameters). • Selection of 5 lesions (≥ 5 × 5 mm) per organ (up to 10 visceral and 5 cutaneous ones). • New lesions are incorporated into the total tumor burden, do not immediately mean progressive disease (PD).
Nishino, M. et al. (2013) [27]	irRECIST	• The immune-related Response Evaluation Criteria in Solid Tumors (irRECIST) criteria. • Unidimensional (longest diameter). • Maximum 5 (2 per organ) lesions (≥ 10 mm in diameter; ≥15 mm for nodal lesions). • New lesions are incorporated in the total measured tumor burden, do not immediately mean PD.
Seymour, L. et al. (2017) [28]	iRECIST	• The immune Response Evaluation Criteria in Solid Tumors (iRECIST) criteria. • Unidimensional (longest diameter). • Maximum 5 (2 per organ) lesions (≥ 10 mm in diameter; ≥15 mm for nodal lesions). • New lesions are recorded separately, not included in the sum of lesions for target lesions identified at baseline. • Defining unconfirmed progressive disease (iUPD) and confirmed progressive disease (iCPD). • iCPD: if additional new lesions appear or an increase in size of new lesions (≥ 5 mm for sum of new target lesion or any increase in new non-target lesion) on next cross-sectional imaging after iUPD.

In recent years, several advanced imaging techniques are available to quantitatively assess tumor status and predict treatment response. The new medical imaging biomarkers change radiology culture towards more quantification and standardization. Beyond RECIST, images including dynamic contrast-enhanced (DCE) computed tomography (CT) or magnetic resonance imaging (MRI), diffusion-weight MR imaging (DWI), magnetic resonance spectroscopy (MRS), ^18^ F-fluorodeoxyglucose (FDG) positron emission tomography (PET) are emerging as promising imaging techniques in the assessment of tumor response to the new anti-cancer drugs [29]. This review evaluates the current RECIST with their limitations and the new emerging concepts of imaging biomarkers in oncology.

### Anatomic or morphological approaches

#### WHO criteria

The WHO established the first standardized approach to evaluate treatment responses of solid tumors based on imaging studies in 1979 [30]. However, the WHO criteria (Table 1) were no longer used after 2000, because of some problems such as the interobserver variability of the number of lesions, selection of measurable targets, minimum lesion size, the definition of progressive disease (PD), and more reliable measures on new imaging technologies had emerged [31].

#### RECIST 1.0 and RECIST 1.1

RECIST guidelines were published in 2000 by an association that comprised the National Cancer Institute of the United States, the European Organization for Research and Treatment in Oncology, and the National Cancer Institute of Canada [3]. The original RECIST version 1.0, provided definitions for “measurable lesion” and “non-measurable lesion” (Table 1). Measurable lesions must have the longest diameter of ≥ 10 mm on CT with a slice thickness of ≤ 5 mm or the longest diameter of ≥ 20 mm on chest radiography [3]. Non-measurable lesions include other lesions that do not meet the criteria as measurable lesions, such as small lesions with a longest diameter less than 10 mm, bone metastases without a soft-tissue component, pleural tumor seeding, lymphangitic tumor spread, and peritoneal or leptomeningeal tumor diseases. After identifying measurable and non-measurable lesions, the RECIST includes the terms “target” and “non-target” lesions. Target lesions are selected based on their size (lesions with the longest diameters) and suitability/conspicuity for accurate repeated measurements. Target lesions include all measurable lesions (up to 5 per organ and 10 in total) that are recorded and measured at baseline. All other lesions were identified as non-target lesions. As several questions emerged, a revised RECIST guideline (RECIST 1.1) was developed in 2009 (Table 1) [4]. Major changes in RECIST 1.1 include the number of target lesions, assessment of pathologic lymph nodes, redefinition of disease progression, clarification of unequivocal progression of non-target lesions, and inclusion of ^18^ F-FDG PET in the detection of new lesions [4]. In RECIST 1.1, the maximum number of target lesions per organ was reduced from 5 to 2, and 10 to 5 in total. Besides, lymph nodes with a short axis ≥ 15 mm were considered measurable, pathological, and assessable as target lesions. Lymph nodes with a short axis < 10 mm were considered normal. Lymph nodes with a short-axis between 10 and 15 mm were identified as non-measurable, non-target lesions. Major changes from the WHO criteria to RECIST 1.1 are summarized in Table 1.

**Fig. 1:**
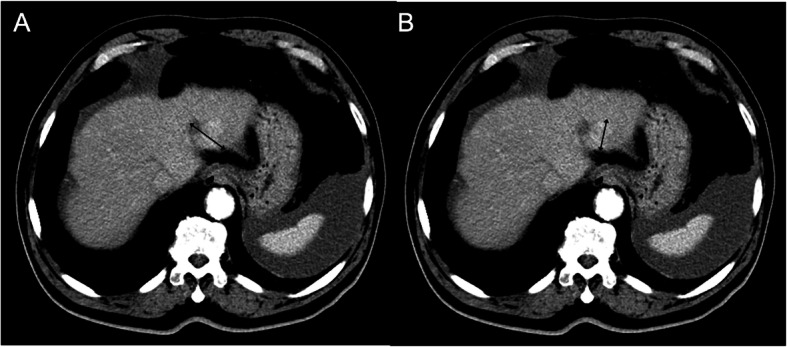
Application of modified Response Evaluation Criteria in Solid Tumors (mRECIST) for hepatocellular carcinoma (HCC). **A** Measurement of the longest overall target tumor diameter (41 mm) according to conventional RECIST. **B** Measurement of the longest viable tumor diameter (30 mm) based on tumor enhancement area on arterial-phase CT imaging according to mRECIST for HCC

**Fig. 2:**
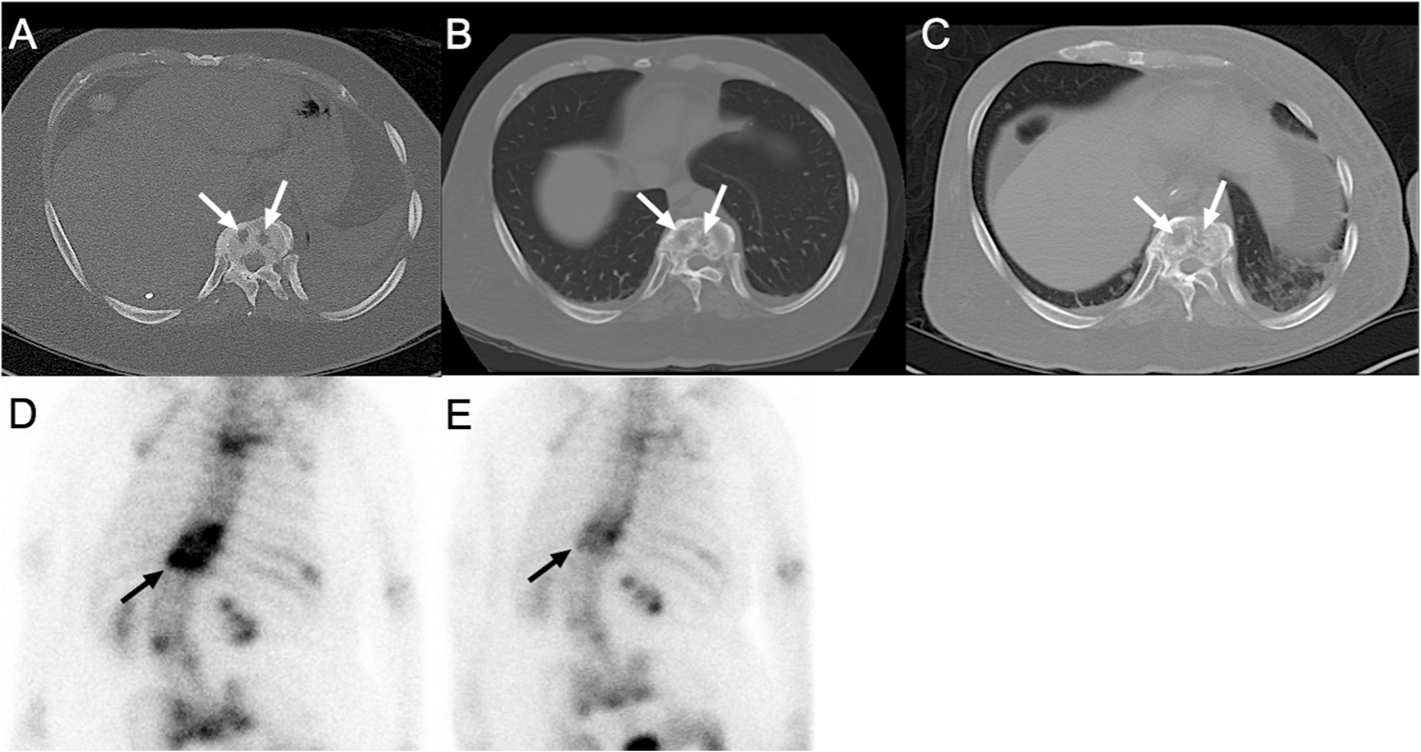
Bone density changes suggest tumor response in bone metastases. A 42-year-old man was diagnosed with lung adenocarcinoma and bone metastases. **A **Pretreatment axial CT scan in the bone window shows two osteolytic metastases (both lesions with diameters of 10 mm) (white arrows) in thoracic vertebrae. **B, C** The osseous lesions have not significantly changed in the sum of longest diameters according to RECIST 1.1 but show osteosclerotic reaction (white arrows) in 6 months (**B**) and 10 months (**C**) after targeted therapy with afatinib, an epidermal growth factor receptor (EGFR) - tyrosine kinase inhibitor (TKI), representing good response. **D, E** Skeletal scintigraphy shows significantly decreased uptake of radiotracer after comparison between pretreatment (**D**) and posttreatment (E) images, which confirmed good therapeutic response

**Fig. 3:**
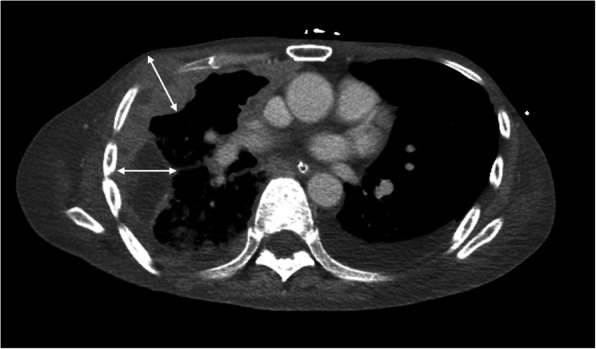
Measurement of tumor thickness for tumor burden assessment in malignant pleural mesothelioma (MPM) according to modified RECIST. The tumor thickness is measured perpendicularly to the chest wall (arrows) or mediastinum, not measuring the tumor longest diameter. The sum of six measured values from two different positions on three different levels is used as modified RECIST in MPM

Evaluation of overall response in RECIST 1.1 is based on tumor responses in target and nontarget lesions (Table 3). For example, if there is any new lesion, the overall response is always PD. In the absence of a new lesion, complete response (CR) is the complete disappearance of all target and nontarget lesions. However, several studies have reported the low reliability of RECIST 1.1 in evaluating treatment response in certain tumors. In HCC, a viable tumor is defined as showing intratumoral arterial enhancement on dynamic CT or MRI. Therefore, measurement of tumor enhancement is used as a surrogate biomarker of a viable tumor (Fig. 1), and tumor necrosis induced by treatment is considered as a response assessment [9]. Several specific criteria other than RECIST for HCC have thus been developed. The criteria include modified RECIST [16] (Table 2), European Association for the Study of the Liver (EASL) criteria [9], and the Response Evaluation Criteria in Cancer of the Liver (RECICL) criteria [32]. The RECIST is also not effective in evaluating treatment response for prostate cancer as there is not enough objective and meaningful measurement of disease progression [10]. In neuro-oncology, RECIST has limited use as the unidimensional measurements do not accurately measure the irregular or asymmetric margins of glioblastoma; besides, it did not take the use of steroids or clinical status into account [11]. The Response Assessment in Neuro-Oncology (RANO) criteria (Table 4) based on McDonald criteria in 2010 contemplated on non-enhancing tumor lesion on T1/T2-weighted imaging and fluid-attenuated inversion recovery (FLAIR) MRI. For bone metastasis, RECIST is limited to measurable metastases or unequivocal progression of the unmeasurable disease. For an accurate response assessment of bone metastases requires visualizing the tumor size as well the structural and metabolic changes in the bone (Fig. 2). This was addressed by the MD Anderson (MDA) bone response criteria (supplemental file) which updated the WHO criteria by expanding radiographic assessment and incorporating both CT and MRI [12, 19]. In MPM, the measurement of the longest unidimensional diameter in tumor mass along the curved chest wall before and after the treatment is difficult that resulted in the development of modified RECIST. The modified RECIST for MPM considers tumor thickness perpendicular to fixed structures such as the chest wall or mediastinum in two positions on the same transverse slice of CT scan, and a sum of six measured values on three different levels was used for evaluation (Fig. 3) [13]. In 1999, an international working group of clinicians, radiologists, and pathologists published Cheson criteria for response assessment and outcomes measurement in lymphoma [33]. Cheson criteria were adopted widely by clinicians and were used in the approval process for several new agents. However, the Cheson criteria were revised in 2007 because of identified limitations and the increased use of ^18^ F-FDG PET, immunohistochemistry, and flow cytometry in lymphoma. For lymphoma, the current standard response criteria are the revised Cheson criteria [20] (Table 5) and Lugano criteria [21] (supplemental file) based on PET or bidimensional tumor measurements on CT for non-FDG avid lesions [21]. In revised Cheson criteria, target lesions are defined based on the different organs (lymph nodes, liver or spleen, other organs) by both CT and PET scans, as well as the clinical examination and bone marrow biopsy. The Lugano classification, published in 2014, eliminated ambiguity and improved evaluations in lymphoma. It included standardized staging criteria for FDG-avid lymphomas using a five-point (Deauville) scale, defined splenomegaly as > 13 cm based on CT image, removed requirements for bone marrow biopsy for routine staging in Hodgkin’s lymphoma and most diffuse B-cell lymphoma, and revised the definitions for PD.

**Table 3:** Assessment of Treatment Response in RECIST 1.1 Criteria

Overall Response	Target Lesions	Non-Target lesions	New lesions
CR	CR	CR	No
PR	CR	Non-CR or non-PD	No
PR	CR	Not evaluated	No
PR	PR	Non-PD or not all evaluated	No
SD	SD	Non-PD or not all evaluated	No
NE	Not all evaluated	Non-PD	No
PD	PD	Any	Yes or No
PD	Any	PD	Yes or No
PD	Any	Any	Yes

*CR* complete response, *NE* not suitable to evaluation, *PD* progressive disease, *PR* partial response, *SD* stable disease

**Table 4:** Response Assessment in Neuro-Oncology (RANO) Criteria

Criterion	CR	PR	SD	PD
T1WI with CE	None	≥ 50 %↓	˂ 50 %↓but ˂ 25 %↑	≥ 25 %↑^a^
T2WI/FLAIR	Stable or ↓	Stable or ↓	Stable or ↓	↑^a^
New lesion	None	None	None	Present^a^
Corticosteroid	None	Stable or ↓	Stable or ↓	N/A^b^
Clinical Status	Stable or ↑	Stable or ↑	Stable or ↑	↓^a^
Response Requirement	All	All	All	Any^a^

*CE *contrast enhancement, *CR* complete response, *FLAIR* fluid attenuated inversion recovery, *PD* progressive disease, *PR* partial response, *SD* stable disease, *T1WI* T1-weighted MR imaging, *T2WI* T2-weighted MR imaging

^a^Progression occurs when any this criterion is present

^b^*N/A* not applicable

**Table 5:** Revised Cheson Criteria for Malignant Lymphoma

Response	Definition	Nodal masses	Spleen, Liver	Bone marrow
CR	Disappearance of all evidence of disease	• FDG-avid or PET positive prior to therapy; mass of any size permitted if PET negative • Variably FDG-avid or PET negative; regression to normal size on CT	Not palpable, nodules disappeared	Infiltrate cleared on repeat biopsy; if indeterminate by morphology, immunohistochemistry should be negative
PR	Regression of measurable disease and no new sites	≥ 50 % decrease in SPD of up to 6 largest dominant masses; no increase in size of other nodes; • FDG-avid or PET positive prior to therapy; one or more PET positive at previously involved site • Variably FDG-avid or PET negative; regression on CT	≥ 50 % decrease in SPD of nodules (for single nodule in greatest transverse diameter); no increase in size of liver or spleen	Irrelevant if positive prior to therapy; cell type should be specified
PD or relapsed disease	Any new lesion or increase by ≥ 50 % of previously involved sites from nadir	Appearance of a new lesion(s) > 1.5 cm in any axis, ≥ 50 % increase in SPD of more than one node, or ≥ 50 % increase in longest diameter of a previously identified node > 1 cm in short axis; lesions PET positive if FDG-avid lymphoma or PET positive before therapy	> 50 % increase from nadir in the SPD of any previous lesions	New or recurrent involvement
SD	Not meet above criteria	• FDG-avid or PET positive prior to therapy; PET positive at prior sites of disease and no new sites on CT or PET • Variably FDG-avid or PET negative; no change in size of previous lesions on CT		

*CR* complete remission, *FDG* fluorodeoxyglucose, *PD* progressive disease, *PET* positron emission tomography, *PR* partial remission, *SD* stable disease, *SPD* sum of the product of the diameters

**Fig. 4:**
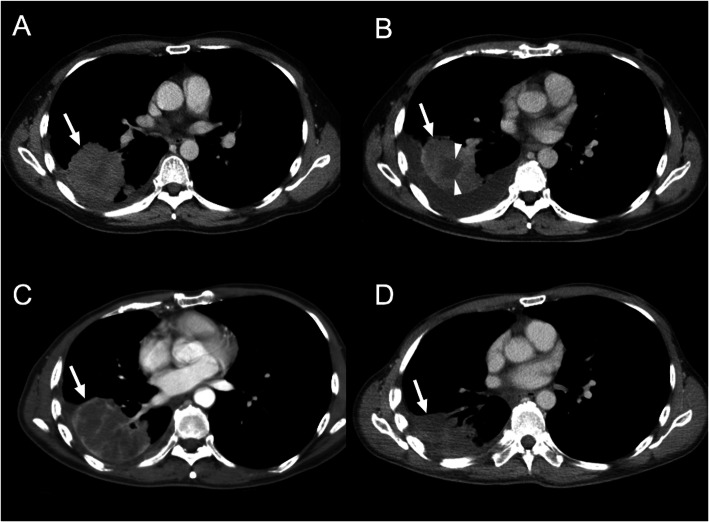
Tumor necrosis indicates early good response in non-small cell lung cancer (NSCLC) receiving targeted therapy. A 53-year-old man diagnosed with NSCLC (positive EGFR exon 20 insertion mutations) and received afatinib therapy. **A** Pretreatment contrast-enhanced (CE) axial CT scan shows an enhancing tumor (arrow), with diameter of 64 mm. **B, C** Pseudo-progression with increased tumor size (arrow) was observed in 3 months (**B**) (67 mm in tumor diameter) and 8 months (**C**) (74 mm in tumor diameter) after targeted therapy. Simultaneously, the progression of focal tumor necrosis (arrowheads in figure **B**) to diffuse tumor necrosis (**C**) was also observed. **D** Shrinkage of tumor mass (50 mm in diameter) was observed 15 months after therapy

In recent years, new cancer immunotherapies such as immune checkpoint inhibitors, especially for advanced non-small cell lung cancer (NSCLC), have drawn greater attention [34]. Due to their peculiar mechanism, immunotherapies can determine unusual response patterns on imaging that cannot be correctly evaluated with the traditional RECIST. Several immune-related response criteria including irRC, irRECIST, and iRECIST were proposed and applied in clinical trials for immunotherapies (Table 2). One of the most common and challenging condition for the morphological evaluation of the response is the “pseudo-progression”, a condition where the target lesion continues to grow at the first imaging study and then remains stable, shrinks in size, or disappears during the subsequent imaging follow-up (Fig. 4). The proposed hypotheses for these morphologic changes include (i) persistent tumor growth during the immune response mounting and/or (ii) the inflammatory process of the existing lesions and other lesions initially not visible on imaging, caused by hyper-activated T-cells [35]. Pseudo-progression has been reported for anti-programmed cell death protein-1 (anti-PD-1), anti-programmed death-ligand 1 (anti-PD-L1), and anti-cytotoxic T lymphocyte-associated antigen 4 (anti-CTLA-4) agents not only in lung cancer but also other cancers, including melanoma, renal cell carcinoma, and bladder cancer [35–37].

#### Special considerations in lung cancer

**Fig. 5:**
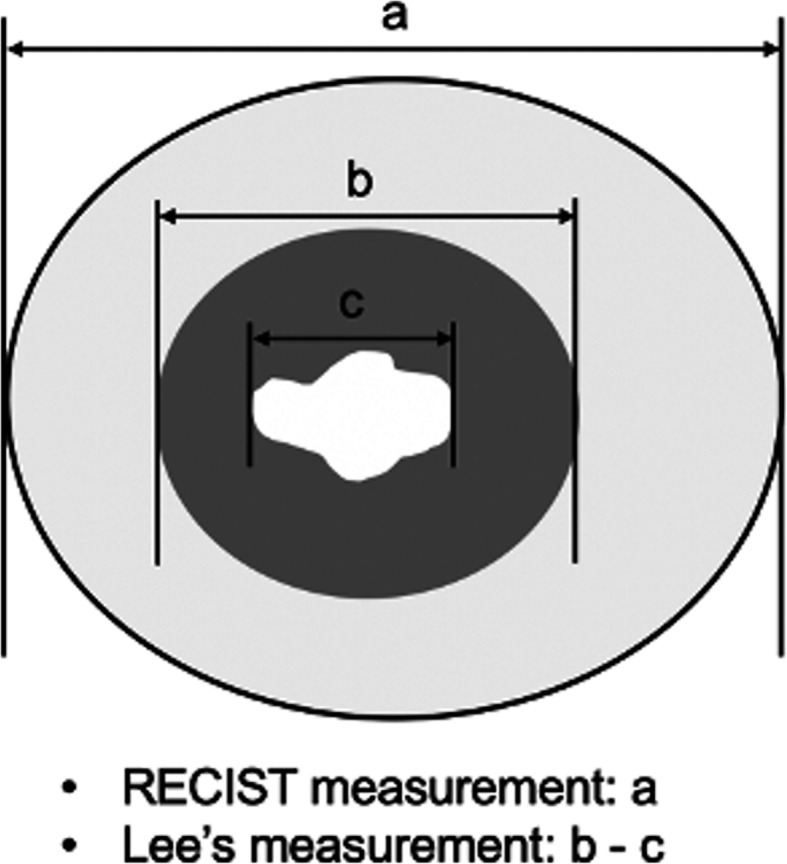
Illustration depicting the target lesion measurement in NSCLC by RECIST and Lee’s criteria. According to RECIST criteria, the size of the target lesion in lung cancer is measured by including both solid and ground-glass opacity (GGO) components (**a**). According to Lee’s criteria, the size of the target lesion is measured by solid component alone on soft tissue window imaging (**b**). If the target lesion has intratumoral cavitation, the size of the target lesion is measured by including only the soft-tissue component and excluding the air component (subtraction of cavity diameter from the longest diameter of tumor mass) (**b - c**)

**Fig. 6:**
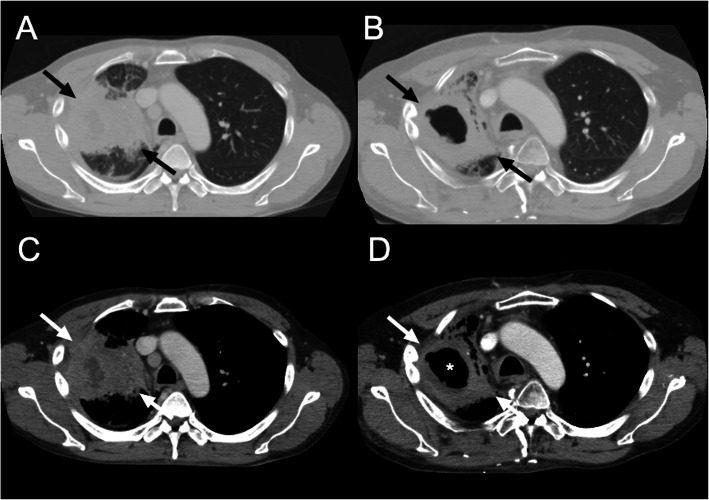
Comparison between RECIST and Lee’s criteria in NSCLC. A 51-year-old man was diagnosed with NSCLC. **A** Pretreatment CE axial CT scan in lung window shows a 92-mm-sized tumor, including both solid and GGO components (black arrows). **B** After targeted therapy with afatinib, posttreatment CE CT scan shows no significant decrease in tumor size (84 mm in diameter, 9 % reduction) (black arrows), suggesting stable disease according to RECIST 1.1. **C** According to Lee’s criteria, the size of the target lesion (white arrows) is measured on pretreatment CE axial CT by solid component alone (79 mm in diameter) on soft tissue window imaging. **D** After targeted therapy, the size of the target lesion is measured by including only soft-tissue tumor (white arrows) (77 mm in diameter) and excluding necrotic air cavitation (asterisk) (49 mm in diameter), thus the tumor size is 28mm (65 % reduction), suggesting good tumor response according to Lee’s criteria

Conventionally, lung cancer size is generally measured on lung window imaging and includes both ground-glass opacity (GGO) and solid components. The size of GGO within lung cancer generally does not vary markedly even after effective chemotherapy. Therefore, size change in the solid component of part-solid lung cancer may be a more accurate reflection of the actual tumor response to anti-cancer chemotherapy. Besides, intratumoral cavitation and necrosis caused by anti-angiogenesis may also indicate tumor response (Figs. 5 and 6). Lee et al. [38] proposed a CT response criterion based on consideration of tumor components (GGO and solid part) and the presence of cavitation, necrosis, and attenuation changes for response assessment in NSCLC patients who underwent epidermal growth factor receptor (EGFR) tyrosine kinase inhibitor (TKI) thertabapy (Figs. 5 and 6).

#### Special considerations in bone metastasis

Bone is one of the most common metastatic sites in lung cancer, breast cancer, or prostate cancer [39, 40]. Treatments for bone metastasis include chemotherapy and endocrine therapy. The treatment response is evaluated by imaging modalities such as radiography, skeletal scintigraphy, CT and MRI, and PET. Accurate response assessment of bone metastases requires evaluation of both the tumor burden and the osteolytic or osteoblastic changes in the bone lesion (Fig. 2). As the RECIST focuses predominantly on the physical measurement of solid tumors, bone metastasis is not easily measured with a ruler and is designated as unmeasurable. Patients with only bone metastases after resection of a primary tumor are not eligible for clinical trials due to no measurable disease. Therefore, the absence of measurable tumors can significantly influence treatments in these patients. The MDA criteria (supplemental file) incorporates information from CT imaging into that of the WHO criteria, specifically for the response assessment in bone metastases. It divides tumor response into 4 standard categories and includes quantitative and qualitative evaluation of the bone metastases. Hamaoka et al. [19] reported that the MDA criteria are superior to the WHO criteria in predicting progression-free survival in breast cancer patients with bone metastasis. In comparison to RECIST 1.1, the MDA criteria allow more metastatic bone lesions to be considered as a measurable disease by allowing physical measurement of well-defined bone lesions regardless of soft tissue extension, allowing subjective assessment of ill-defined lesions, and considering healing sclerosis. Other functional imaging criteria such as Positron Emission Tomography Response Criteria in Solid Tumors (PERCIST) (Table 6) allow bone metastases to be measured by assessing tumor metabolism in the absence of anatomic change [12].

**Table 6:** Positron Emission Tomography Response Criteria in Solid Tumors (PERCIST)

Response	Criteria
CR	• Disappearance of all metabolically active tumor
PR	• > 30 % decrease in SUL peak (minimum 0.8 unit decrease) in lesion with greatest uptake (not necessarily the same lesion)
PD	• > 30 % increase in SUL peak (minimum 0.8 unit increase) • > 75 % increase in total lesion glycolysis • New lesions
SD	• Does not meet above criteria

*CR* complete metabolic response, *PD* progressive metabolic disease, *PR* partial metabolic response, *SD* stable metabolic disease, *SUL* standardized uptake value using lean body mass

### Clinical limitations and challenges of morphological evaluation

According to RECIST, the standard method to assess the response of solid tumors to chemotherapy is to decide those target lesions first and perform a uni-dimensional measurement of tumor size. For patients with multiple lesions, the selection of targeted lesions in different organ may be different among the operators. Linear measurements of tumor size may have limitations related to variability in technical and imaging factors, tumor enhancement and morphology, and reader decisions [41]. These factors result in the challenge for the comparison of tumor size change over time. Tumor size changes between interval studies, including true tumor changes and concomitant variations or errors in measurement. Variability can be caused by scan-rescan variability and both intra- and inter-observer variability between two repeat readings of the same scan. Oxnard et al. [42] reported that changes of tumor size less than 10 % can be a result of the inherent variability in patients with advanced NSCLC. This variability was the greatest in the measurement of small tumors and the significant importance for the accurate determination of disease progression.

The measurement of the entire tumor volume overcomes some of the limitations, as to improve the ability to reliably detect small changes in measurements, and to increase statistical power per subject in trials [43]. Neither the RECIST nor the WHO criteria include volume measurement because of limitations in past diagnostic imaging techniques and the available measurement methods. But with the advent of thin-section CT and commercially available tumor segmentation software, it is now possible to obtain image data sets with spatial resolutions adequate to measure tumor volumes [44]. Zhao et al. [45] suggested that measuring volumetric changes in tumor dimension may hold the potential to be an earlier biomarker of tumor regression or progression. The changes in tumor volume may be assessed as early as 3 weeks after the initiation of gefitinib (Iressa) treatment, whereas a lower magnitude of changes in unidimensional and bidimensional measurements was seen during the same period [45].

### Functional, metabolic, and other non-morphological approaches

Functional and metabolic imaging techniques can integrate pathological, physiological, and morphological changes, and serve as potential early predictors for therapeutic response [24, 46]. The microscopic changes in the tumor microenvironment are detected and early evaluation of response to therapy such as tumor attenuation/enhancement, perfusion, oxygenation, metabolism, etc., are possible.

#### Evaluation of tumor attenuation/enhancement

**Fig. 7:**
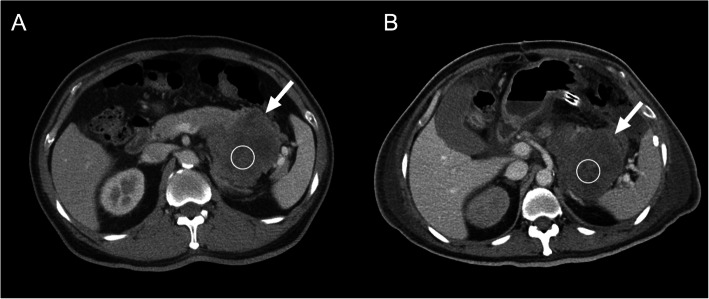
Application of Choi criteria in the gastrointestinal stromal tumor (GIST) after targeted therapy with imatinib. A 49-year-old man was diagnosed with GIST. **A** Pretreatment CE axial CT scan shows an 88-mm-sized enhancing tumor (arrow) arising from the stomach. The measured CT number on the region of interest (ROI) is 36.1 Hounsfield units (HU). **B** After targeted therapy with imatinib, posttreatment CE CT scan shows no significantly decreased in tumor size (87 mm in diameter) (arrow) but markedly decreased attenuation (22.6 HU, 37 % reduction), suggesting tumor response according to Choi criteria

Tumor enhancement/attenuation changes in CT are important imaging biomarkers for the assessment of tumor responses [22, 47, 48]. In patients receiving molecular targeting agents, a decrease of tumor attenuation on CT indicates a response to therapy, even in the absence of decreased tumor size as defined by RECIST. In contrast, tumor progression may demonstrate patterns of new intratumoral enhancing lesions rather than tumor size increase. Choi criteria [22] (Table 7) were the first to introduce this CT parameter for evaluating the effect of imatinib mesylate (Gleevec), a TKI, in gastrointestinal stromal tumors (GIST). For malignant GISTs, early dramatic changes in the extent of tumor attenuation are often observed after using imatinib (Fig. 7) [49]. However, morphological changes in unmodified RECIST alone may not be sufficient to estimate the effect of imatinib in GISTs, especially at an early stage of treatment that are poor predictors of clinical benefit. Choi criteria define a good response by a 10 % decrease in tumor size or a 15 % decrease in CT attenuation (Table 7). Choi criteria also define PD by the (1) appearance of new lesions, (2) appearance or increase in the size of intratumoral nodules, or (3) tumor size increase by more than 20 % without post-treatment hypoattenuation change.

**Table 7:** Choi Criteria

Response	Criteria
CR	• Disappearance of all lesions • No new lesions
PR	• A decrease in tumor size ≥ 10 % or a decreased in tumor attenuation (Hounsfield unit) ≥ 15 % on CT • No new lesions • No obvious progression of non-measurable disease
PD	• An increase in tumor size ≥ 10 % and does not meet criteria of PR by tumor attenuation on CT • New lesions • New intratumoral nodules or increase in size of the existing intratumoral nodules
SD	• Does not meet criteria of CR, PR, or PD • No symptomatic deterioration resulted from tumor progression

*CR* complete response, *PD* progressive disease, *PR *partial response, *SD* stable disease

Similar phenomena were observed in other solid tumors, including HCC, colorectal liver metastases, renal cell carcinomas (RCC), and sarcomas [22, 47, 48]. In 2000, the EASL agreed that estimating the reduction in viable tumor volume (recognized as poorly-enhanced areas on dynamic CT or MRI) should be considered for locoregional therapies such as trans-arterial chemoembolization (TACE) or radiofrequency ablation (RFA) in HCC [9]. In HCC, several studies showed a poor correlation between the clinical benefits and conventional response criteria in treatments by sorafenib and locoregional therapies such as TACE or RFA [50, 51]. Therefore, a modified RECIST (mRECIST) was described by Lencioni et al. [16] in 2010. The mRECIST for HCC was developed based on the concept that a viable tumor is defined as showing intratumoral arterial enhancement by contrast agent during dynamic CT or MRI (Fig. 1) [9]. Therefore, measurement of tumor enhancement can be used as a surrogate biomarker of a viable tumor. Furthermore, tumor necrosis induced by treatments should be considered as a response assessment. Besides, for patients with colorectal liver metastasis, tumor attenuation correlates well with survival rate after bevacizumab treatment, whereas RECIST criteria do not correlate with patient survival [52]. For metastatic RCC treated with sunitinib, the Choi criteria also had a better predictive value than RECIST in defining patients who benefit from therapy [53].

#### CT and MRI perfusion imaging

The basis for using CT and MRI perfusion in oncology is that the microvascular changes of angiogenesis are reflected by neovascularization in tumor cells [54]. These techniques can quantify regional tumor blood flow (BF), tumor blood volume (BV), micro-vessel permeability, contrast medium extraction fraction, and extraction fraction on plasma and interstitial volumes. Clinical applications of CT and MRI perfusion include lesion characterization, tumor staging, predicting therapeutic response and clinical outcome, and evaluation of tumor relapse and drug resistance [55].

The degree of CT perfusion is related to tumor aggressiveness in many types of cancer, and highly vascularized tumors are associated with a poor prognosis [54, 56, 57]. Higher baseline BF on CT perfusion predicts better response to chemotherapy and radiotherapy in different tumors. Bellomi et al. [58] reported that patients with higher baseline BF and BV had better responses in rectal carcinoma. In squamous cell carcinoma of the head and neck, Hermans et al. [59] showed that lower CT perfusion values had a significantly higher local failure rate. Hypervascular tumors allow delivery of chemotherapy and may have more oxygenation for greater radiosensitivity [60, 61]. Different antiangiogenic agents such as antibodies targeting vascular endothelial growth factor (VEGF) and small-molecule TKIs have been developed [57, 62]. Higher pretreatment CT perfusion values are associated with a better response rate to antiangiogenic drugs [63, 64]. As compared to RECIST and tumor attenuation, Jiang et al. [65] proposed that CT perfusion is a more sensitive imaging biomarker in advanced HCC patients treated with a combination of anti-angiogenic and conventional chemotherapies.

**Fig. 8:**
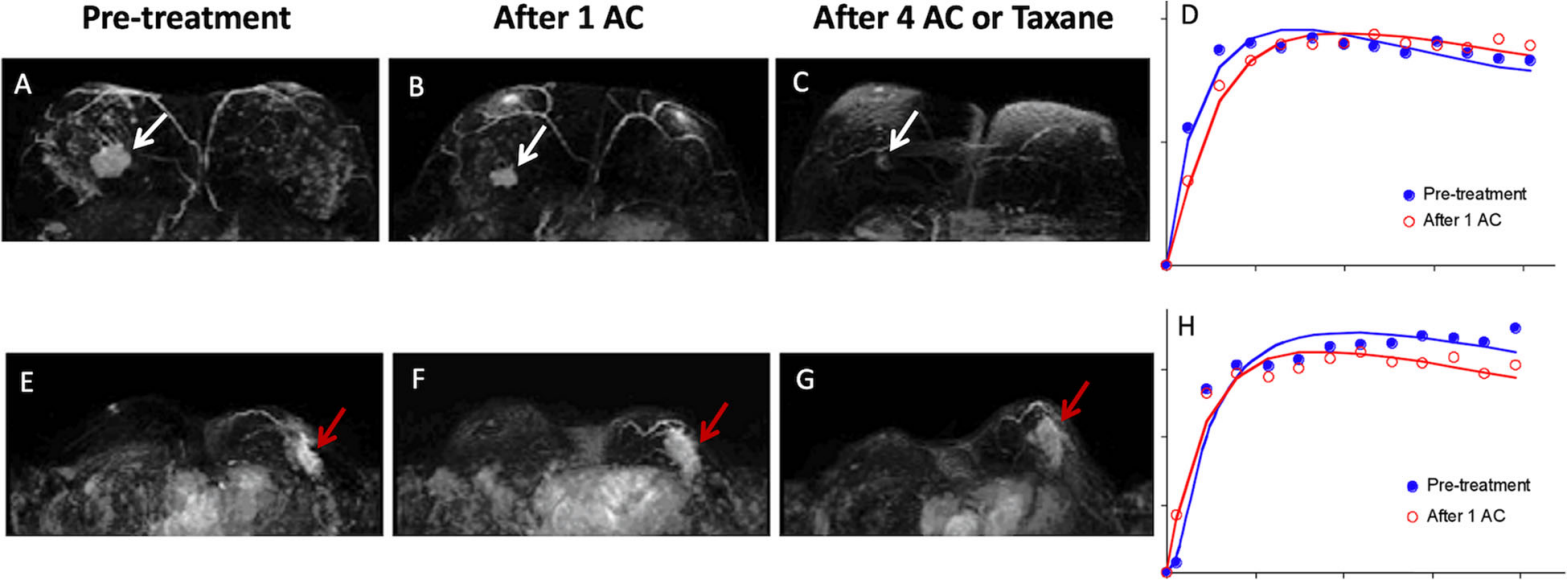
MRI-dynamic contrast-enhanced (DCE) kinetic curve can be used to predict chemotherapy response. **A - C** The upper panel shows a woman with breast cancer in the right breast. The tumor responded very well to chemotherapy. After one cycle of Adriamycin and Cyclophosphamide (AC), and 4 cycles of AC or taxane, the tumor size was remarkably reduced (white arrows). **D** Note the change of the DCE kinetic curves, acquired from the pretreatment MRI (blue) and after 1 cycle of AC (red), from washout pattern to more flattened pattern, indicating the malignant cells were being eliminated. **E - G** The lower panel is a woman of non-responder. The breast cancer in the left breast was not reduced in size following chemotherapy (red arrows). **H** Note the DCE kinetic curve acquired from MRI after 1 cycle of AC (red), became more apparently a washout pattern compared with the pre-treatment curve (blue)

MRI perfusion also has the ability to analyze underlying tumor angiogenesis by parameters related to tumor perfusion and permeability after the intravenous injection of gadolinium-based contrast agents [66]. Depending on the technique utilized, MRI perfusion can provide information about tissue cerebral blood volume and perfusion (using dynamic susceptibility contrast-enhanced [DSC] MRI), or micro-vessel permeability and the extracellular space (using T1-weighted dynamic contrast-enhanced [DCE] MRI). DSC-MRI is usually used for the evaluation of brain tumors, and the most commonly calculated parameters are relative cerebral blood volume (rCBV), relative cerebral blood flow (rCBF), and mean transit time (MTT). Pharmacokinetic analysis of DCE-MRI is the most widely used method for the quantitative measurement of vessel permeability changes (Fig. 8). With the Tofts model [67], an evaluation of the injected contrast agent leaking into the extravascular-extracellular space, and tissue perfusion and permeability becomes possible. The volume transfer constant K^trans^ (wash-in rate; unit: min^− 1^) describes the forward leakage rate of the contrast medium. For blood vessels where the leakage is rapid, perfusion determines the contrast agent distribution and K^trans^ is similar to tissue blood flow per unit volume [68]. This is typically reported in breast tumors where the endothelium is leaky and other high permeability situations and is termed a flow-limited situation. For the prediction of therapeutic efficacy in tumors, both blood perfusion and micro-vessel permeability are important determinants [69]. Morabito et al. [69] reported that both blood perfusion (rCBV) and micro-vessel permeability (K^trans^) are useful tools for differentiating tumor recurrence from radiation necrosis in brain tumors. Quantitative assessment of contrast enhancement kinetic curve on breast DCE-MRI resulted in excellent diagnostic performance for differentiation of malignancy (Fig. 8) [70]. Wedam et al. [71] reported that DCE-MRI parameters including K^trans^, kep (reverse rate constant from tumor to the vascular space), and ve (extravascular volume fraction) could be used as an early imaging biomarker for monitoring treatment response in breast cancer patients receiving bevacizumab. Other cancers that can be monitored by DCE-MRI include radiotherapy in rectal cancer [72], metastatic renal cell carcinoma treated with sorafenib [73], androgen withdrawal therapy in prostate cancer [74], and radiofrequency ablation in HCC [75]. It is known that successful treatment usually leads to decreases in BF and permeability in assessing response to chemotherapy, radiotherapy, and anti-angiogenic therapy [76].

#### Diffusion-weighted MR imaging (DWI)

The consensus concerning DWI as a cancer imaging biomarker was reached in 2008 at the International Society for Magnetic Resonance in Medicine Meeting [77]. Therefore, DWI has been widely used as an imaging biomarker in the characterization of malignancy, determination of lesion aggressiveness, and monitoring response to a variety of treatments [77–79]. In theory, DWI provides biomedical information related to tissues and structures of interest-based on the measurement of thermally induced “Brownian motion” of water molecules shown by apparent diffusion coefficient (ADC) value [80]. The information provided by DWI includes tissue perfusion, cellularity, extracellular space distribution, and the integrity of cell membranes. Thus, unusual imaging findings on DWI could be an early predictor of biophysiological abnormality [77]. In oncologic imaging, DWI has been linked to tumor aggressiveness and treatment response [77]. Parameters derived from DWI are appealing as imaging biomarkers because the acquisition is rapid and noninvasive, requiring no exogenous contrast agents or exposure to ionizing radiation.

In oncologic practice, the measurements of water diffusivity are used as biomarkers of tissue properties. Since tissue water movements are not ‘‘free’’ but impeded by cells, extracellular matrix, and other molecules, the measurement of tissue water diffusivity is often termed the ADC value. The biomarkers used in oncology include relative signal intensity at different b-values, water diffusivity (D), perfusion fraction (F_p_), ADC_total_, fractionated ADC (ADC_fast_ and ADC_slow_), and fractional anisotropy (FA) [81]. F_p_ represents the contribution of blood microcirculation and microscopic flow to signal decay. ADC_fast_ is calculated using low b-values (0-100 s/mm^2^) and is dominated by this perfusion component of the total tissue diffusivity. At higher b-values (> 100 s/mm^2^), the perfusion component is largely extinguished, so ADC_slow_ measurements are more heavily determined by water diffusion within the cellular matrix. For most clinical studies, only ADC_total_ is reported, which is the total ADC including perfusion contributions. In the above discussion, it is assumed that water movements in tissues are nondirectional (isotropic). However, in some tissue such as brain white matter and renal tubules, water mobility in normal tissues can be directional (anisotropic). A commonly used measurement of anisotropy is FA, which is a dimensionless quantity with range between 0 (nondirectional, isotropic) to 1 (highly directional, anisotropic) [82].

**Fig. 9:**
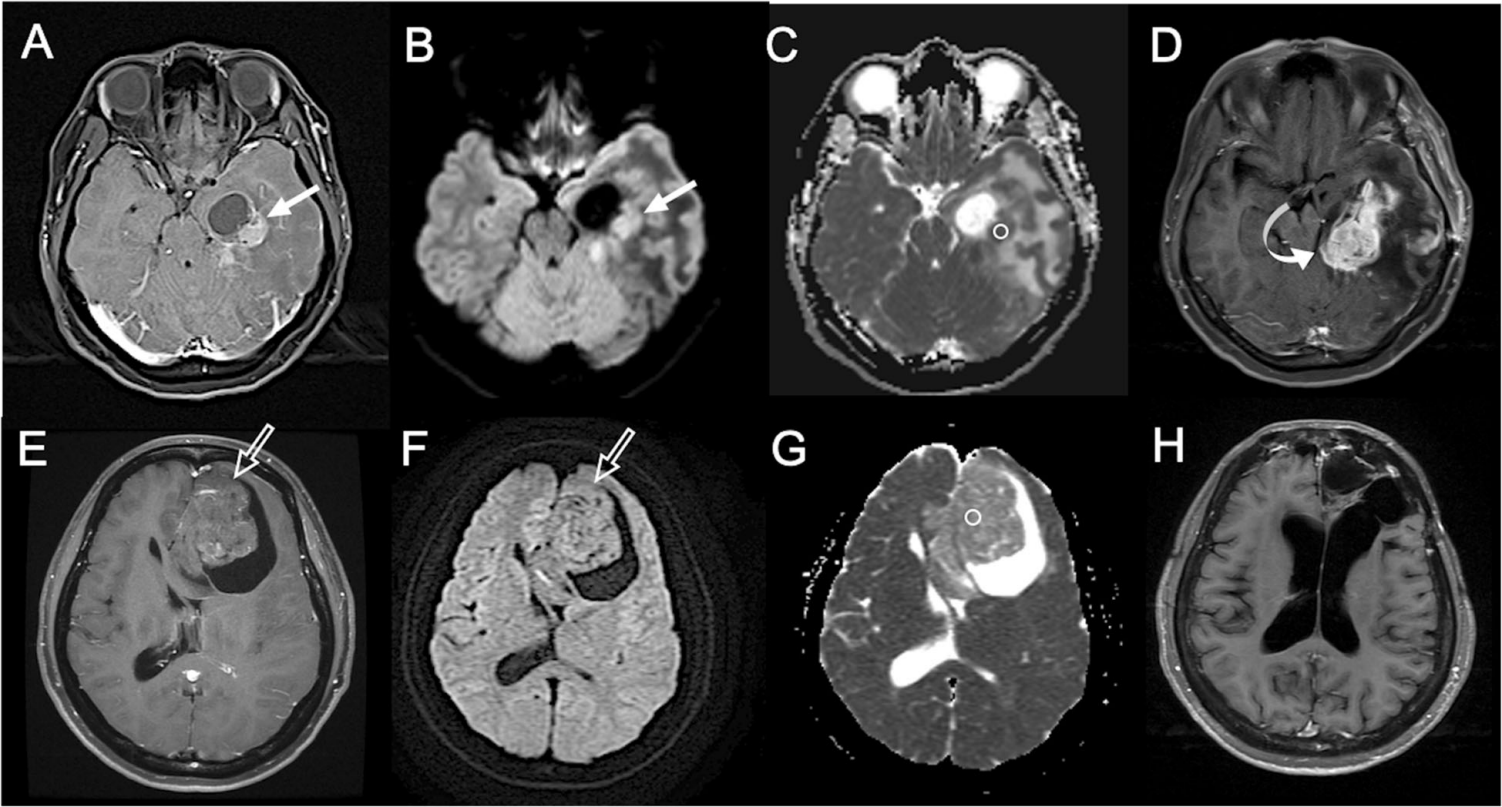
Low apparent diffusion coefficient (ADC) value on diffusion-weighted imaging (DWI) can predict worse therapeutic response in brain glioblastoma. **A** Pretreatment CE axial T1-weighted imaging (T1WI) shows a 40-mm-sized enhancing glioblastoma (arrow) with the cystic component in the left temporal lobe. **B** The DWI shows hyperintensity in the solid part of the tumor (arrow), indicating a diffusion restriction phenomenon. **C** The measured ADC value (b = 1000 s/mm^2^) on ROI is 0.72 × 10^− 3^ mm^2^/sec. (D) Rapid tumor recurrence (tumor diameter of 54 mm) (curved arrow) was observed 3 months after surgical resection. **E** Pretreatment CE axial T1WI shows another 65-mm-sized enhancing glioblastoma (open arrow) with a cystic component in the left frontal lobe. **F** DWI shows isointensity (no diffusion restriction) in the solid part of the tumor (open arrow). **G** The measured ADC value on ROI is 1.42 × 10^− 3^ mm^2^/sec. (H) No tumor recurrence was observed 72 months after surgical resection

The DWI offers useful clinical information at all stages of cancer, including detection, diagnosis, staging, monitoring therapy response, assessing recurrence, and developing pharmaceutical drugs [77]. The applications of DWI as an early biomarker for the prediction of treatment outcomes in different tumors such as brain glioblastoma, hepatic tumor, lung cancer, and primary bone sarcomas had been reported (Fig. 9) [83–87]. Pretreatment low ADC values correlate with poor survival in malignant brain astrocytomas independent of tumor grade (Fig. 9) [88]. Higher pretreatment ADC values were associated with better therapeutic response in vascular disruptive agents [89]. In contrast, several clinical studies have noted that higher pretreatment ADC values respond less favorably to treatments [90–92], reflecting the presence of microscopic and macroscopic necrosis, which is recognized to be associated with poorer therapeutic outcomes [93]. Association between necrosis and hypoxia in tumors is probably involved. Hypoxia in tumor cells mediates resistance to chemotherapy, radiation, and photodynamic therapy [93]. The association between higher pretreatment ADC values and the poorer response had been reported in rectal cancer, liver metastasis, and breast cancers [94–96]. Furthermore, successful treatment is reflected by increased ADC values in several different tumors, including breast cancer, HCC, malignant brain tumors, and primary bone sarcomas [97–100].

#### Magnetic Resonance Spectroscopy (MRS)

The alterations in metabolite levels are best detected both noninvasively and quantitatively by MRS. Although MRS is usually applied in brain lesions, it can also be used for detection, staging, evaluation of aggressiveness, and assessment of therapeutic response in the breast, prostate, hepatic, and other cancers [46]. MRS can be procured from several nuclei in the body, such as 1-Hydrogen (1 H), 31-Phosphorus, and 19-Fluorine. Typically, 1 H-MRS is the most common clinically used method because of the high sensitivity to this nucleus, easy accessibility, and the abundant existence of hydrogen in metabolites. MRS procedure begins with the acquisition of MR images and then assesses the “metabolite spectrum” in the region of interest. In single voxel spectroscopy (SVS), a single voxel (volume of tissue) is located in the tumor or lesion where the metabolism may be damaged as a result of patient disease. Another technique of MRS is known as chemical shift imaging (CSI) [101]. In CSI, a large volume divided into several smaller voxels is selected to produce all voxel’s spectrum simultaneously. Usually, SVS is the preferred choice when accurate quantification is required, and CSI is used to provide an overall vision of spatial distributions of the metabolite [102, 103].

In brain tissue, N-acetyl aspartate (NAA), creatine (Cr), and choline (Cho) are the most important metabolites as an MRS signal [104]. The derivatives of Cr form an important system for energy metabolism, and their intensity is considered fixed. Therefore, they are applied in the computing of metabolite ratios. Brain tumors commonly cause an increase in Cho concentration. Cho level is related to the ability of proliferation and existence of malignancy, and an increased Cho signal is a marker of the presence of a brain tumor [105]. In contrast to Cho, the signal of NAA, which is a neuronal marker, typically decreases in brain tumors. Since the level of Cho and NAA signal changes in brain tumors, the calculation of this Cho-NAA ratio is helpful in the interpretation of brain tumor’s MRSI spectra [106]. The anaerobic metabolism in tumor cells also causes the appearance of lactate peak in the brain tumor’s spectra. The increase in lactate and lipid peaks are the markers of tumor progression and transformation from a low-grade to a high-grade tumor [107].

**Fig. 10:**
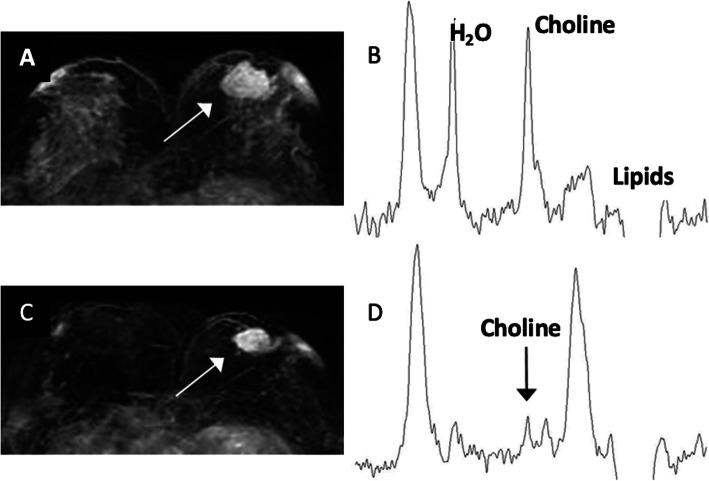
Proton magnetic resonance spectroscopy (MRS) can early predict chemotherapy response. **A** A woman with 34 mm breast cancer in the left breast (white arrow). **B** The pretreatment MRS shows a choline peak of 2.33 mmol/kg. **C** After one cycle of Adriamycin and Cyclophosphamide, the tumor was 26 mm (white arrow), showing a 24 % reduction in size. According to the RECIST 1.1, this is a non-responder. **D** However, posttreatment MRS shows much more sensitive evidence of tumor response with a 51 % reduction of total choline level (from 2.33 mmol/kg to 1.15 mmol/kg) (black arrow)

For breast cancer, Roebuck et al. [108] first proposed that the Cho peak can be applied as a sign of malignancy (Fig. 10). Several studies also showed that Cho peak is a useful marker in malignant breast lesions, and it is not visible in normal tissues or benign tumors [109, 110]. Further, Jagannathan et al. [111] first reported the Cho peak is diminished in 89 % of cases that underwent chemotherapy. These promising results suggest that MRS increases the sensitivity, specificity, and accuracy of breast MRI and will be a useful tool in the diagnosis and management of breast cancer [112]. In prostate cancer, CSI of MRS has shown that it has high diagnostic accuracy [113]. The MRS gathers metabolic information of prostatic tissue by calculating the relative concentrations of citrate, Cr, and Cho. Further, prostate adenocarcinoma can be differentiated from normal adjacent tissue based on the (Cho + Cr)/citrate ratio with a cut-off value of 0.8 [114, 115]. H1-MRS can also be applied in the diagnosis of gastrointestinal tumors and malignant ovarian tumors [116, 117]. These results revealed that most cancer lesions can be determined by the increase of Cho and lactate peaks with a decrease of lipid peak [118].

#### Metabolic imaging

**Fig. 11:**
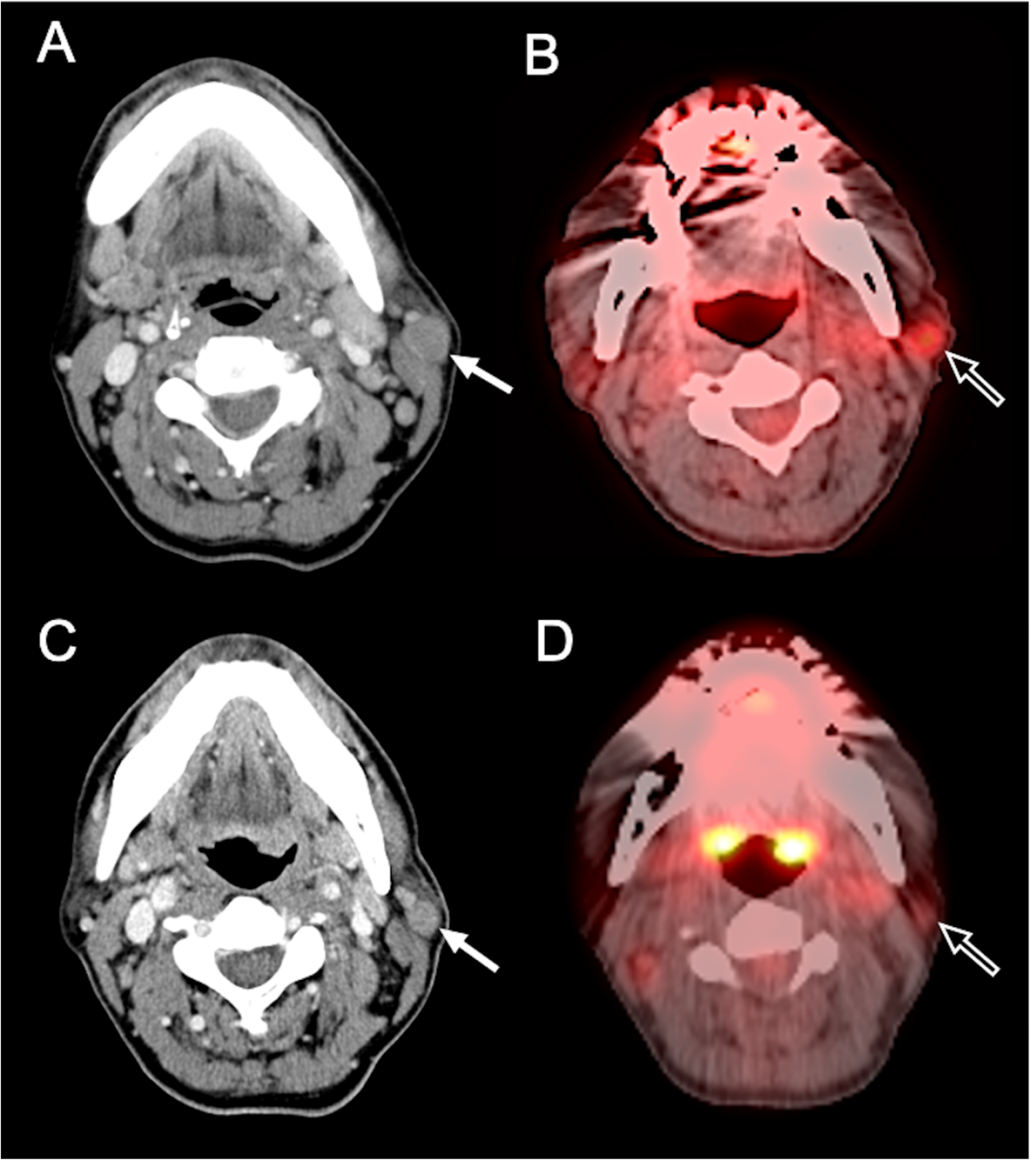
^18^ F-fluorodeoxyglucose (FDG) positron emission tomography (PET) can predict early response in targeted therapy. A 53-year-old man was diagnosed with Hodgkin lymphoma at the left parotid gland. **A** Pretreatment CE axial CT imaging shows a 16 mm tumor mass at the left parotid gland (white arrow). **B** The SUVmax value of 3.0 in the target lesion (open arrow) was detected on a pretreatment PET-CT scan. **C** After five cycles of brentuximab vedotin, a tumor size of 13.5 mm (white arrow) was observed, showing a 16 % reduction in size. According to the RECIST 1.1, this is a non-responder. **D** However, posttreatment PET-CT scan shows good tumor response with 50 % reduction of SUVmax value (from 3.0 to 1.5) (open arrow)

For newer anti-cancer therapies that stabilize disease rather than reduce tumor size at the beginning of treatments, the ^18^ F-FDG PET offers particularly valuable information in these cases. PET can assess tissue metabolism by using radiolabeled molecules, most commonly ^18^ F-FDG, a glucose analog. ^18^ F-FDG PET-CT or MRI is of value in the differentiation of benign and malignant tissues, preoperative staging, recurrent disease detection, and identification of early tumor response to therapy. Wahl et al. [24] proposed guidelines for the standardization of response criteria for FDG PET, the so-called PERCIST criteria (Table 6). The standardized uptake value (SUV) normalized by lean body mass (SUL) represents a quantitative assessment of uptake in a tumor region of interest. The SUV/SUL is based on a ratio between tracer uptake within a tumor and homogeneous distribution of tracer within the patient body. By reflecting the change in tumor metabolism, ^18^ F-FDG PET establishes a method to measure tumor response in the absence of marked anatomic changes (Fig. 11) [119]. A decrease in FDG uptake indicates treatment response or improves survival rates in patients with solid tumors such as breast cancer [120], esophageal cancer [121], lung cancer [122, 123], osteosarcoma [124], and other tumors [125].

^18^ F-FDG PET provides more rapid response data than morphological measurements [126, 127]. In patients with NSCLC, ^18^ F-FDG PET is a staging tool, and the SUV provides prognosis before and after the treatment [122, 123]. It prevented futile thoracotomies in lung cancer [128] and stratified patients into surgical versus palliative groups in colorectal cancer [129]. However, the SUV changes are also influenced by other factors such as vascular delivery. In primary breast tumors, a reliable drop-in SUVs indicating a tumor response is seen only for patients with high initial SUVs [130]. Therefore, a reduction in PET metabolism caused by chemotherapy may be dependent on pre-therapy vascular delivery [131].

#### Radiomics approach

Radiomics is a possible exciting complement to RECIST for monitoring and predicting therapeutic response. Both CT and MRI radiomics analysis are new field in medical imaging. This approach is based on computerized extraction of several quantitative imaging features, and uses these data for the medical decision, prediction, and monitoring response to therapy [132]. A general pipeline of radiomics analysis includes feature extraction, feature selection, and prediction. Feature extraction quantitatively defines the imaging parameters from the specified areas of the images. Feature selection evaluates the feature importance based on the objectives, and the prediction model is established by selected features [132]. Radiomics extracts a large number of quantitative imaging features from a medical image and then analyses these features by a series of machine learning algorithms. The extracted imaging features are related to the underlying anatomical microstructure and biophysical processes such as genetic expression, tumor proliferation, and tumor neovascularization [133]. Radiomics in texture and shape analysis had been widely used to evaluate medical images with promising results [134, 135]. Recently, radiomics analysis is emerging as a comprehensive quantitative method to diagnose and evaluate response to therapy in brain tumors, head and neck cancer, breast cancer, liver tumor, prostate cancer, rectal cancer, non-small-cell lung cancer, and metastatic RCC [136–143]. With radiomics, texture analysis reveals visually imperceptible information that extends beyond radiology to histopathology and provides predictors for diagnosis, prognosis, and therapeutic planning for cancer patients [136–139].

Radiomics is especially a possible useful tool to predict response to radiation therapy, different chemotherapies, and immune checkpoint inhibition treatments, especially in lung cancer [144, 145]. It can be potentially integrated into the normal clinical workflow to identify lung cancer patients who would benefit most from therapy [144]. Huynh et al. [145] reported that CT-based radiomic shape and tumor heterogeneity features could predict the treatment response to stereotactic body radiation therapy in early-stage NSCLC. In locally advanced NSCLC, Rakshit et al. [146] showed that certain textural features are radiomics predictors for response to pemetrexed chemotherapy, with an AUC of 0.81. Velcheti et al. [147] developed a pretherapy CT radiomics-based predictive model for the prediction of tumor response to Nivolumab in locally advanced NSCLC, with an AUC of 0.84. Other studies applying radiomics on NSCLC also showed very positive and promising results [148, 149].

## Conclusions

Conventionally, tumor response is evaluated basically and readily by the use of RECIST 1.1. However, the criteria mainly focus on tumor dimensional changes and do not reflect other functional, metabolic, and non-morphological changes that may occur in molecular-targeted therapy, immunotherapy, and local treatments. Since morphological changes in unmodified RECIST 1.1 alone may not be sufficient to estimate the treatment response in cancer patients, several modified criteria have been proposed to improve the response assessment in different specific tumors. Due to the rapid progress in anti-cancer therapies nowadays, the new imaging techniques such as CT/MRI-based perfusion, DWI, MRS, PET, and radiomics should be recognized by radiologists and oncologists for tumor response evaluation. On the other hand, although imaging is utilized for evaluating tumor response in daily oncological practice, the situation in which imaging is used differs to some degree between clinical trials and routine clinical practice [150]. The challenges of standardizing imaging in the multi-center clinical trials include the processes of image acquisition, data analysis, and radiological review [150]. The emergence of new treatment paradigms and the trend toward personalized treatment should be accompanied by the evolution of response assessment. Although these new imaging techniques make clinically effective contributions to cancer care as a decision-making method for personalized medicine [151], it may be cost prohibitive and challenging to implement in some institutions at this moment. To resolve this problem, the aim toward developing more affordable and effective alternatives is necessary. In our opinion, integrating multiparametric quantitative information from different imaging modalities for precise personalized medicine and evaluation is the goal of cancer treatment in the future

## Supplementary Information

**Additional file 1.**

## Data Availability

The datasets used during the current study are available from the corresponding author on reasonable request.

## Abbreviations

RECIST: Response Evaluation Criteria in Solid Tumors
CT: Computed tomography
MRI: Magnetic resonance imaging
DWI: Diffusion-weight MR imaging
MRS: Magnetic resonance spectroscopy
FDG: ^18 ^F-fluorodeoxyglucose
PET: Positron emission tomography
WHO: World Health Organization
HCC: Hepatocellular carcinoma
MPM: Malignant pleural mesothelioma
DCE: Dynamic contrast-enhanced
PD: Progressive disease
EASL: European Association for the Study of the Liver
RECICL: Response Evaluation Criteria in Cancer of the Liver
RANO: Response Assessment in Neuro-Oncology
FLAIR: Fluid-attenuated inversion recovery
MDA: MD Anderson
NSCLC: Non-small cell lung cancer
anti-PD-1: Anti-programmed cell death protein-1
anti-PD-L1: Anti-programmed death-ligand 1
anti-CTLA-4: Anti-cytotoxic T lymphocyte-associated antigen 4
GGO: Ground-glass opacity
EGFR: Epidermal growth factor receptor
TKI: Tyrosine kinase inhibitor
PERCIST: Positron Emission Tomography Response Criteria in Solid Tumors
GIST: Gastrointestinal stromal tumors
RCC: Renal cell carcinoma
TACE: Trans-arterial chemoembolization
RFA: Radiofrequency ablation
mRECIST: Modified RECIST
BF: Blood flow
BV: Blood volume
VEGF: Vascular endothelial growth factor
DSC: Dynamic susceptibility contrast-enhanced
rCBV: Relative cerebral blood volume
rCBF: Relative cerebral blood flow
MTT: Mean transit time
ADC: Apparent diffusion coefficient
F_p_: Perfusion fraction
FA: Fractional anisotropy
SVS: Single voxel spectroscopy
CSI: Chemical shift imaging
NAA: N-acetyl aspartate
Cr: Creatine
Cho: Choline
SUV: Standardized uptake value
SUL: Standardized uptake value normalized by lean body mass
