# Supplementary material for: Imaging biomarkers for evaluating tumor response: RECIST and beyond
Source: Biomark Res. 2021 Jul 2;9:52. doi: 10.1186/s40364-021-00306-8 (PMC8252278; doi:10.1186/s40364-021-00306-8)
Supplement: Supplementary file 1 — Additional file 1. [file 40364_2021_306_MOESM1_ESM.docx]

**MD Anderson (MDA) Criteria^a^**

| Response category | Criteria |
| --- | --- |
| CR | - Complete sclerotic fill-in of lytic lesions on XR or CT - Normalization of bone density on XR or CT - Normalization of signal intensity on MRI - Normalization of tracer uptake on SS |
| PR | - Development of a sclerotic rim or partial sclerotic fill-in of lytic lesions on XR or CT - Osteoblastic flare - Interval visualization of lesions with sclerotic rims or new sclerotic lesions in the setting of other signs of PR and absence of progressive bony disease - ≥ 50% decrease in measurable lesions on XR, CT, or MRI - ≥ 50% subjective decrease in the size of ill-defined lesions on XR, CT, or MRI - ≥ 50% subjective decrease in tracer uptake on SS |
| PD | - ≥ 25% increase in size of measurable lesions on XR, CT, or MRI - ≥ 25% subjective increase in the size of ill-defined lesions on XR, CT, or MRI - ≥ 25% subjective increase in tracer uptake on SS - New bone metastases |
| SD | - No change - < 25% increase or < 50% decrease in size of measurable lesions - < 25% subjective increase or < 50% subjective decrease in size of ill-defined lesions - No new bone metastases |

^a^ Measurements are based on the sum of a perpendicular, bidimensional measurement of the greatest diameters of each individual lesion. CR: complete response, CT: computed tomography, MRI: magnetic resonance imaging, PD: progressive disease, PR: partial response, SD: stable disease, SS: skeletal scintigraphy, XR: radiography

**Lugano Criteria**

| Modality | CT | ^18^F-FDG PET |
| --- | --- | --- |
| CR | - Lymph nodes ≤ 1.5cm in Ldi - Complete disappearance of radiologic evidence of disease | - Scores 1, 2, 3 in nodal or extranodal sites with or without a residual mass |
| PR | - Single lesion: ↓ ≥ 50% in PPD - Multiple lesions: ↓ ≥ 50% in SPD of up to six lymph nodes or extranodal sites | - Scores 4 or 5 with ↓ uptake compared with baseline and residual mass |
| SD | - ↓ ≤ 50% in SPD of up to six lymph nodes or extranodal sites (no criteria for PD are met) | - Scores 4 or 5 with no obvious change in FDG uptake |
| PD | - New lymphadenopathy or↑; single node must be abnormal with : a) Ldi > 1.5cm and b) PPD ≥ 50% and c) Ldi or Sdi↑0.5 cm if ≤ 2cm and↑1.0 cm if > 2cm - ↑splenic volume: a) with prior splenomegaly:↑> 50% of its prior↑beyond baseline; b) without prior splenomegaly:↑> 2.0 cm; c) new or recurrent splenomegaly - New or larger non-measured lesions - Recurrent previously resolved lesions - New extranodal lesion > 1.0 cm in any axis (new lesions < 1.0cm in any axis are included if attributable to lymphoma) - A new node > 1.5 cm in any axis | - Scores 4 or 5 in any lesion with↑uptake from baseline - New FDG-avid foci |

CR: complete remission, FDG: fluorodeoxyglucose, Ldi: longest transverse diameter, PD: progressive disease, PET: positron emission tomography, PPD: product of perpendicular diameters; PR: partial remission, SD: stable disease, Sdi: shortest transverse diameter, SPD: sum of the product of the diameters
